# Using blood data for the differential diagnosis and prognosis of motor neuron diseases: a new dataset for machine learning applications

**DOI:** 10.1038/s41598-021-82940-8

**Published:** 2021-02-09

**Authors:** Alberto Greco, Maria Rosa Chiesa, Ilaria Da Prato, Anna Maria Romanelli, Cristina Dolciotti, Gabriella Cavallini, Silvia Maria Masciandaro, Enzo Pasquale Scilingo, Renata Del Carratore, Paolo Bongioanni

**Affiliations:** 1 Department of Information Engineering, Faculty of Engineering, University of Pisa, Pisa, Italy; 2 Institute of Clinical Physiology Research, CNR-Pisa, Pisa, Italy; 3 Translational Medicine Dpt, University of Pisa, Pisa, Italy; 4 Severe Acquired Brain Injuries Dpt Section, Azienda Ospedaliero-Universitaria, Pisa, Italy; 5 NeuroCare onlus, Pisa, Italy

**Keywords:** Neurological disorders, Biomarkers

## Abstract

Early differential diagnosis of several motor neuron diseases (MNDs) is extremely challenging due to the high number of overlapped symptoms. The routine clinical practice is based on clinical history and examination, usually accompanied by electrophysiological tests. However, although previous studies have demonstrated the involvement of altered metabolic pathways, biomarker-based monitoring tools are still far from being applied. In this study, we aim at characterizing and discriminating patients with involvement of both upper and lower motor neurons (i.e., amyotrophic lateral sclerosis (ALS) patients) from those with selective involvement of the lower motor neuron (LMND), by using blood data exclusively. To this end, in the last ten years, we built a database including 692 blood data and related clinical observations from 55 ALS and LMND patients. Each blood sample was described by 108 analytes. Starting from this outstanding number of features, we performed a characterization of the two groups of patients through statistical and classification analyses of blood data. Specifically, we implemented a support vector machine with recursive feature elimination (SVM-RFE) to automatically diagnose each patient into the ALS or LMND groups and to recognize whether they had a fast or slow disease progression. The classification strategy through the RFE algorithm also allowed us to reveal the most informative subset of blood analytes including novel potential biomarkers of MNDs. Our results show that we successfully devised subject-independent classifiers for the differential diagnosis and prognosis of ALS and LMND with remarkable average accuracy (up to 94%), using blood data exclusively.

## Introduction

Motor neuron diseases (MNDs) represent a heterogeneous group of lethal neurodegenerative disorders whose causes are still largely unknown^1^. The annual incidence is 2 per 100,000 and prevalence of 5–7 per 100,000^2,3^. MND leads to progressive muscle weakness and atrophy, with upper or lower motor neuron signs, or a mixture of them, due to the degeneration of pyramidal neurons in the motor cortex, cranial motor neurons, and anterior horn cells in the spinal cord. Amyotrophic lateral sclerosis (ALS) is the most common expression of the disease. It involves both upper and lower motor neuron symptoms. Less common variants are a pure upper motor neuron disease (UMND, primary lateral sclerosis), or a pure lower motor neuron disease (LMND, progressive muscular atrophy)^4^.

Even if the diagnosis of MND is correct in 95% of the cases, the absence of a specific diagnostic test makes it difficult to distinguish ALS from other MNDs with the selective involvement of the upper or the lower motor neuron^1,5^, and to find specific therapeutic markers for different MND types^6^. Indeed, ALS can come with a prognostic outlook often ambiguous and undistinguishable compared to other MNDs^5^. Recently, brain MRI studies have shown fast-progressing LMND as a possible ALS phenotypic variant, whereas slow-progressing LMND patients have been considered phenotypically different from ALS patients^7,8^ (even if a clear and unique progression rate (PR) threshold has not been yet identified^7,9,10^). In addition, symptoms may vary among individuals and, especially at the early stages of the disease, an in-depth neurological exam may have similar results for different MNDs, making the differential diagnosis hard to apply. Particularly, neurologists often fail to make a diagnosis of ALS as compared to other MNDs within the first year of illness^11^. The long latency in the differential diagnosis of most ALS/MND cases limits the possibility of a proper therapeutic approach^12,13^. In contrast, an earlier diagnosis reduces the period of uncertainty for the patient allowing them to plan the future care and the essential support, which may have an impact on the progression of the disease^3^.

The current formal diagnosis of ALS is clinical and based on the revised El Escorial criteria^4,14–16^. Other tools such as neuroimaging, electrophysiology, and cerebrospinal fluid (CSF) have the limited role of excluding the possibility of alternative neurological conditions with similar symptoms^17^. Previous studies on blood data have already proven the involvement of altered metabolic pathways in MNDs, despite limiting their investigation on ALS patients, i.e., the most common form of MND^18–22^. However, biomarker-based monitoring tools are still far from being applied in the clinical practice^23^. More specifically, Lu et al.^23^ have already evaluated the combined blood expression of neuromuscular and inflammatory biomarkers as predictors of disease progression and prognosis in ALS. Furthermore, ALS-specific systemic inflammatory signals have also been reported, including a reduced frequency of regulatory T cells in the blood in individuals with a faster disease progression^18–22^. A common limitation of studies investigating MND-related blood analytes is due to the small number of analytes that are usually arbitrarily and heuristically chosen. A more innovative way to proceed might start from a bigger number of blood parameters later selected according to a data-driven strategy.

In this work, we introduce a new dataset containing diachronic clinical and biochemical data acquired over the last 10 years from both ALS and LMND patients. Each patient has been clinically followed up by the same experienced neurologist through periodical medical examinations and blood analysis until either today or his/her death. Our dataset is unique in the scientific literature as every single record combines clinical outcomes with a remarkable collection of 108 common and rare blood analytes, including haemochrome indexes, haemostasis and metabolism parameters, routine functional profiles of the main organs, and inflammatory/immunological and oxidative markers.

Through the application of robust and well-validated statistical and machine-learning (ML) methods to this new dataset, we aim to detect specific patterns of blood analytes capable of automatically discriminating ALS from LMND patients helping out in the prognosis.

ML techniques have already been successfully applied to ALS data sets and some promising diagnosis models have been proposed^17^. Prognostic models have been tested using clinical, biological, and neuroimaging data^17^. However, to the best of our knowledge, there are no studies that have applied ML techniques to support a differential diagnosis of different MNDs. The main limitation of classification performance is due to the small number of training samples compared to the large number of features. In our study, we have addressed the issue of the poor sample-to-feature ratio by successfully applying a feature selection algorithm that uses a backward elimination procedure^24,25^. Thanks to this method, we identified the smallest but at the same time most informative subset of blood analytes with the aim of reducing the necessary number of blood analyses and, consequently, increasing the cost-effectiveness.

## Methods

### Standard protocol approvals, registrations, and patient consents

The ethical approval was obtained from the Tuscany Ethics Committee $\hbox {N}^{\circ }$ 14568. All participants signed an informed consent or, if this was not possible, gave their verbal permission for a carer to sign on their behalf. Moreover, all methods were carried out following relevant guidelines and regulations.

### Patient recruitment criteria

#### Inclusion criteria

Our study included 726 blood samples acquired from 41 ALS and 25 LMND patients, diachronically withdrawn during the last 10 years. Among these, we considered 692 blood samples acquired from 35 ALS and 20 LMND patients to build the dataset and the classifiers presented in this work. The remaining 34 blood samples acquired from 6 ALS and 5 LMND additional patients were included in the study at a later stage only for performance evaluation of the classification analyses (see “Generalization performance evaluation” section).

All ALS and LMND patients underwent periodical electrophysiological examinations including electromyography, electroneurography, and motor/magnetic evoked potentials. The patients were diagnosed and included in the study according to the El Escorial revised criteria^4,15^. According to these criteria, the ALS patients showed the simultaneous presence of upper (cortical) and lower (brainstem or spinal) motor neuron signs such as spastic tone, hyperreflexia, clonus, pathologic reflexes; the indisputable progression of the disease; the absence of an alternative reasonable explanation for symptoms and signs. On the other hand, LMND criteria considered only patients with the exclusive presence of lower motor neuron signs combined with weakness, muscle atrophy, fasciculations, and the indisputable progression of the disease. Of note, we enrolled only “clinically definite” patients, namely those with clinical signs of the involvement of both the upper and lower motor neurons for the ALS group or of the exclusive involvement of the lower motor neuron for the LMND group, in three out of four body regions: bulbar, cervical, thoracic, and lumbosacral. More in detail, we enrolled in the study 14 ALS and 6 LMND patients showing clinical signs in the bulbar, cervical, and lumbosacral regions; 7 ALS and 3 LMND patients in the bulbar, cervical, and thoracic regions; 5 ALS and 5 LMND patients in the bulbar, thoracic and lumbosacral regions; and 16 ALS and 11 LMND patients in the cervical, thoracic and lumbosacral regions.

It is important to note that LMND patients in the course of their disease could exhibit symptoms and signs related to the involvement of the upper motor neuron, and consequently fall within the diagnosis of ALS. Accordingly, we included in our study only LMND patients who kept exclusive involvement of the lower motor neuron over time: i.e., patients who were diagnosed with LMND one year or longer prior the study and did not manifested any impairment of the upper motor neuron in the meantime. In addition, during the study, they were re-evaluated, and only those still meeting the criteria for “clinically definite” LMND were definitively considered.

#### Exclusion criteria

We excluded patients suffering from “clinically probable” or “clinically possible” ALS or LMND, namely those with clinical signs in one or two body regions only, respectively. Moreover, we excluded patients suffering from UMND and those clinically definite, probable, and probable-laboratory supported ALS or LMND also suffering from other neurological diseases (cerebrovascular, neuroinflammatory/immune or neurodegenerative), and/or severe brain injuries, and/or severe non-neurological illnesses (cardiovascular and blood diseases, kidney, liver or pancreas failure, immune disorders).

### Data collection

Over the last 10 years, we have collected 692 clinical and blood data from 35 ALS and 20 LMND patients approximately every 3 months. The data have been used to developing an on-going database including symptom onset (defined as the first patient-reported body weakness complaint^23^, PR, and other clinical data, together with 108 blood analytes (Table 1).

#### Clinical data

Clinical data include demographics, medical history, treatment information, and disease severity index. This latter was scored according to the revised form of the ALS Functional Rating Scale, ALSFRSR^26^. In addition, we calculated the disease PR by subtracting the ALSFRSR score from 48 (i.e. the maximal ALSFRSR score) and dividing by the disease duration (from the symptom onset) expressed in months^23^. Within both our ALS and LMND groups of patients, we considered two sub-groups according to their PR. Specifically, we defined a relatively slower progressing sub-group and a relatively faster-progressing sub-group using a cut-off of 0.5 as in^23^. Accordingly, within the LMND dataset, 185 blood data were labeled as “low PR” and 99 as “high PR”. Instead, concerning the ALS group, blood samples were divided into two groups of 259 and 149 data with low land high PR, respectively.

#### Lab data

Blood analytes (n=108) included haemochrome and routine profiles for kidney, liver, pancreas, and heart functions, together with haemostasis and metabolism parameters, inflammatory and immunological markers (lymphocyte subsets, immunoglobulins, cytokines and growth factors), and oxidative markers, which are thoroughly reported in Table 1.

#### Database description

We considered three different datasets: one including all the 692 clinical and blood data from both patient groups (*all-patients*), and two sub-datasets selecting only patients at their early disease stages, namely those with high scores ($\ge \,35/48$) of ALSFRSR (*hSc*) and those within their first year from the symptom onset (*1-y*). More in detail, the *hSc* dataset represents a group of data taken from patients both with benign prognosis (from the clinical outcome) and at the beginning of their disease course. This included 44 patients (30 ALS and 14 LMND) for a total of 143 blood samples. The *1-y* dataset included 31 patients (20 ALS and 11 LMND) for a total of 70 blood samples acquired during the first year of the course of the disease, without considering the prognosis. Comparison between *hSc* /*1-y* ALS and *hSc* /*1-y* LMND might help us to get information for an early differential diagnosis.

Data are available upon reasonable request and verification of all ethical aspects, at p.bongioanni@ao-pisa.toscana.it.

**Table 1:** List of all analytes with the related acronyms and the group they belong to.

**Inflammation and Immunology**		MON	Absolute monocyte count	**Cell development and survival**	
APO1/FAS	Apoptosis antigen-1/FAS	MON%	Monocytes percentage	EGF	Epidermal growth factor
BAS	Absolute basophil count	NEU	Absolute neutrophil count	EPO	Erythropoietin
BAS%	Basophils percentage	NEU%	Neutrophils percentage	FGF	Fibroblast growth factor
CD16+56	CD16+56+ lymphocytes	PAlb	Prealbumin	IGF1	Insulin growth factor 1
CD19	CD19+ lymphocytes	SAA	Serum amyloid A	PDGF	Platelet-derived growth factor
CD25	CD25+ lymphocytes	TNF$\alpha$	Tumor necrosis factor-alpha	TGF$\beta$1	Transforming growth factor beta 1
CD3	CD3+ lymphocytes	TNF$\alpha$R1	Tumor necrosis factor-alpha receptor I	VEGF	Vascular endothelial growth factor
CD4	CD4+ lymphocytes	TNF$\alpha$R2	Tumor necrosis factor-alpha receptor II	**Oxidative stress**	
CD40R	CD40 receptor	WBC	White blood cells	FRD	Free radical derivatives
CD45	CD45+ lymphocytes	**Cell adhesion**		GPx	Glutathione peroxidase
CD45RA	CD45RA+ lymphocytes	ICAM1	Intercellular adhesion molecule 1	GR	Glutathione reductase
CD45RO	CD45RO+ lymphocytes	MMP9	Matrix metalloproteinase 9	SOD	Superoxide dismutase
CD8	CD8+ lymphocytes	Sel E	Selectin E	TPAO	Total plasma antioxidants
CRP	C reactive protein	Sel L	Selectin L	**Metabolism**	
EOS	Absolute eosinophil count	Sel P	Selectin P	ALT	Alanine aminotransferase
EOS%	Eosinophils percentage	VCAM1	Vascular cell adhesion molecule 1	Amy	Amylase
ESR	Erythrocyte sedimentation rate	**Basics**		AST	Aspartate aminotransferase
Fibr	Fibrinogen	$\alpha$1GI	Alpha1 globulin	BA	Biliary acids
$\gamma$Gl	Gamma globulin	$\alpha$2GI	Alpha2 globulin	Bil	Bilirubin
Ifn$\beta$	Interferon beta	Alb	Albumin	Chol	Total cholesterol
IgA	Immunoglobulin A	$\beta$1GI	Beta1 globulin	CK	Creatine kinase
IgE	Immunoglobulin E	Ca	Calcium	Cre	Creatinine
IgG	Immunoglobulin G	Cl	Chloride	Fe	Iron
IgM	Immunoglobulin M	Hb	Hemoglobin	Fer	Ferritin
IL1	Interleukin 1	Hct	Hematocrit	Fol	Folate
IL2	Interleukin 2	INR	International normalized ratio	GGT	Gamma glutamyltransferase
IL3	Interleukin 3	K	Potassium	Glu	Glucose
IL4	Interleukin 4	MCH	Mean corpuscle hemoglobin	HDL	High-density lipoprotein Chol
IL5	Interleukin 5	MCHC	Mean corpuscle hemoglobin content	LA	Lactic acid
IL6	Interleukin 6	MCV	Mean corpuscle volume	LDH	Lactic dehydrogenase
IL7	Interleukin 8	Mg	Magnesium	Lip	Lipase
IL8	Interleukin 10	Na	Sodium	Tran	Transferrin
IL6	Interleukin 12	P	Phosphorus	Trig	Triglicerids
IL2R	Interleukin-2 receptor	Plt	Platelet	Urea	Urea
IL6R	Interleukin-6 receptor	PT	Prothrombin time	VitB12	Vitamin B12
LYM	Absolute lymphocyte count	PTT	Partial thromboplastin time		
LYM%	Lymphocytes percentage	PTTr	Partial thromboplastin time ratio		
MCP1	Monocyte chemoattractant protein	RBC	Red blood cells		

### Statistical and classification analysis

The dataset comprising of 692 observations and 108 features, and its subsets described in “Data collection” section, were used to perform exploratory statistical analysis and to build five different pattern recognition systems.

#### Descriptive statistics

An exploratory group-wise statistical comparison between ALS and LMND patients was performed for each blood analyte. We used a non-parametric Mann-Whitney U test with a Holm-Bonferroni adjustment for multiple testing. The same non-parametric statistical analysis was used also to analyze possible statistical differences between both ALS and LMND patients with low (< 0.5) and high PR ($\ge$ 0.5).

#### Classification analysis

For each of the three datasets described in “Database description” (i.e., *all-patients*, *hSc*, and *1-y*), we performed a classification analysis aiming at distinguishing between the ALS and LMND groups using only blood data information. Moreover, a further classification analysis was performed on the complete dataset only to distinguish, within each of the two groups, between patients with high PR and low PR.

**Figure 1:**
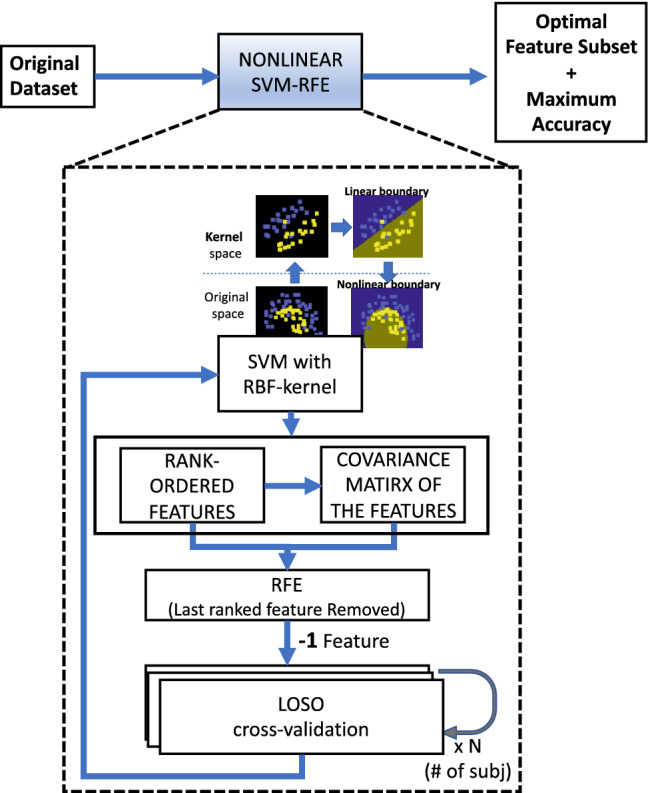
SVM-RFE with correlation bias reduction—conceptual scheme.

Our learning algorithm is based on a support vector machine (SVM) model. The SVM finds the decision boundary that maximizes the margin separating the two classes of training data points. However, due to the characteristics of our dataset, two main issues needed to be addressed: first, our data were not linearly separable, i.e., the boundary between the two classes could not be linear as in standard SVM; secondly, the very high number of features (i.e., analytes) compared to the number of data points led to a high overfitting risk, as well as less interpretable results. To solve the first issue, we adopted an RBF kernel that mapped the original input dataset into a new space where our data became linearly separable (using the “kernel trick”) (see Fig. 1). Alternatively, we can say that the RBF kernel made our decision boundary nonlinear. To address the second point, we employed a feature selection (FS) strategy. Particularly, we implemented a recently developed recursive-feature-elimination (RFE) algorithm embedded in the SVM model, including also a correlation bias reduction strategy^27^. Embedded FS ranked the features based on their importance in separating the two classes through a specific classifier, i.e., the SVM. Once we ordered the features, we iteratively removed the last ranked since it has the least effect on classification. At each iteration step, we estimated the classification performance (i.e., accuracy) until all the features have been removed (Fig. 1). The later a feature was removed, the more important it was.

The classifier model was fit and evaluated through a leave-one-subject-out procedure (LOSO) which is a nearly unbiased estimator of the out-of-sample error^28–30^. More in detail, within the LOSO scheme, considering N subjects, iteratively we split the feature-set into a training set, comprising of *n* observations from ($\hbox {N}-1$) patients, and into a test set comprising of the *m* observations from the remaining patient. This approach is indeed a highly reliable procedure, especially in the case of multiple correlated observations from the same source^31^.

To solve the SVM optimization problem, we used the default hyper-parameters and solver suggested by LIBSVM library^32^. Indeed, when FS algorithms are adopted, they already lead to a deep exploration of the hypothesis space. Therefore, a parameter tuning might often lead to an over-searching condition with consequent over-optimistic accuracy estimation, as well as a high computational cost.

In summary, the employed method combined both the possibility of a nonlinear model and an FS strategy that also mitigates the bias due to correlated features^27^. Particularly, FS had a crucial role not only to maximize the classification accuracy and reduce the overfitting risk, but also to allow us to remove the irrelevant, noisy, and redundant analytes highlighting the most informative subset^9,33^. Previous studies have proved that embedded FS, i.e., scoring features based on the output of a predictive model, commonly outperform the other FS strategies such as Filter and Wrapper approaches^9,33^. Of note, further embedded approaches for reducing the dimension of the feature space were tested, e.g. LASSO-based models such as L1-SVM and LASSO binomial generalized linear model. However, very poor results were achieved, probably because L1-regularization does not enable employing the RBF kernel, which has proved to play a crucial role in the good classification of our datasets.

#### Generalization performance evaluation

As mentioned in “Lab data”, to measure the classifier generalization performance, we recruited 11 additional patients (6 ALS and 5 LMND) to build an independent test set comprised of 34 new blood samples. These patients were included in the study only at the end of the model identification analyses to estimate the generalization error in an unbiased way. Since this test set did not include patients at the early stage of the disease, it was used to test the generalization performance of only three classifiers: (i) ALS versus LMND considering the *all-patients* dataset, (ii) High versus Low PR considering the ALS dataset, and (iii) High versus Low PR considering the LMND dataset. It is worthwhile noting that, unlike the training and validation sets, such a test set included only the reduced subset of analytes previously selected through the LOSO validation procedure.

## Results

In this section, we present the results obtained from both statistical analysis and classification. The section is organized into sub-sections according to the kind of comparison (ALS versus LMND or high PR versus Low PR), the dataset considered, and the kind of analysis (statistics and classification).

### ALS versus LMND statistical comparison

#### *All-patients* dataset

Concerning the dataset that includes the whole group of patients, we observed that most of the analytes’ average values were within the normal healthy ranges for both ALS and LMND (except for IGF1, MMP9, ICAM1, VCAM1, and IgE). Instead, the results of the statistical comparison revealed several analytes that significantly differed between ALS and LMND.

The list and relative descriptive statistics of the significant analytes (corrected p value $< 0.05$) are shown in Table 2. The most relevant differences are described hereafter. Specifically, ALS had a significantly inferior quantity of RBC, but in a larger size (MCV) and containing more Hb (MCH, MCHC) than LMND. Chol and Trig blood content was also higher in the LMND group as well as the number of growth factors (FGF and IGF1) and cell-adhesion molecules (ICAM1 and VCAM1). Instead, ALS patients showed a higher level of Fe and Fer (associated with a reduced amount of Tran), and higher values of CK (associated with lower values of LA) than LMND. From the inflammatory-immunological analyte group, ALS patients had a lower amount of alpha- and beta-globulin, but a higher number of gamma-globulins as well as IgG, IgA, IgM, and IgE content. Other relevant immunological biomarkers were found significantly different between the two patients’ groups: ALS showed higher amounts of CD3 and CD4, IL3, IL8, and lower levels of CD19 lymphocytes, soluble IL2R, soluble IL6R, TNF, TNFRs, and IL12 (Table 2).

#### *hSc* and *1-y* datasets

When we consider only the subset of patients with high ALSFRSR (i.e., *hSc* group), we note that ALS patients had average lower values of Tran and higher levels of iron than LMND ones. Moreover, also in this case, several immunological biomarkers were found significantly different between the two patients’ groups (see Table 2): results revealed a higher percentage of CD4 cells and IL3 as well as a significantly lower percentage of CD8 and CD25 cells in the *hSc*-ALS group. Focusing on the *1-y* dataset, *1-y*-LMND patients had a significantly higher level of soluble IL2R than *1-y*-ALS patients.

### ALS versus LMND classification results

**Figure 2:**
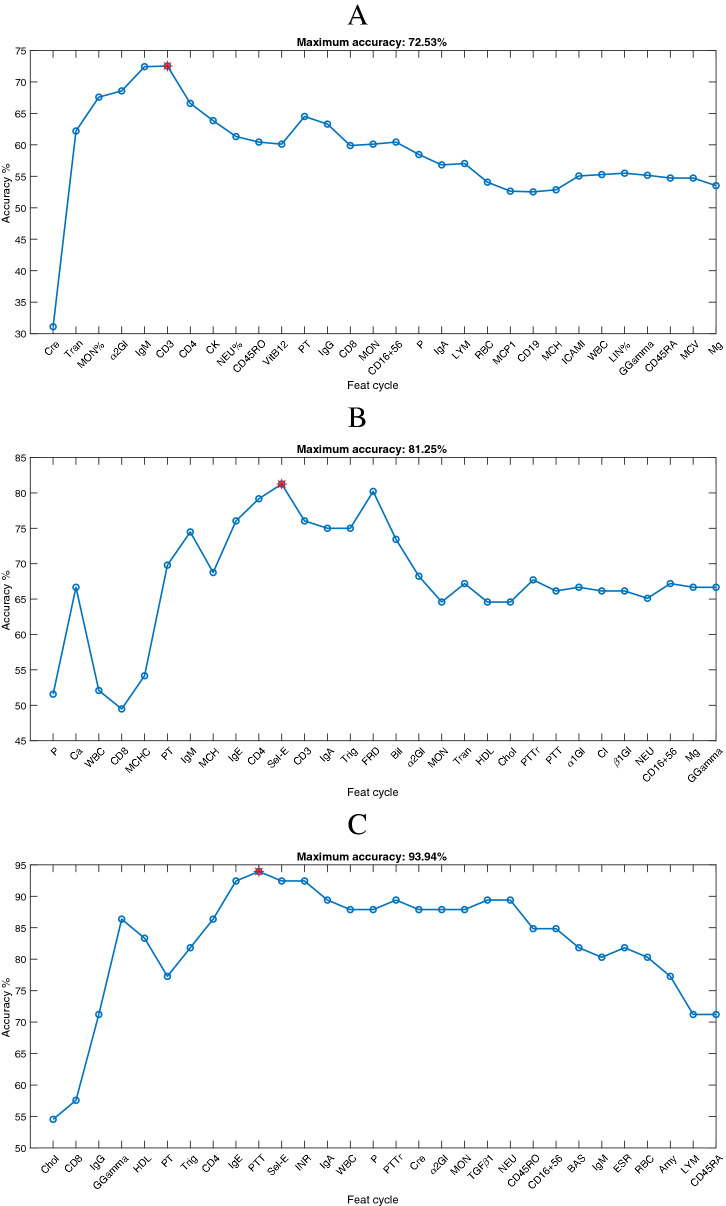
Classification accuracy trend of the ALS versus LMND recognition model as a function of the first 30 selected blood analytes. The red marker indicates the maximum accuracy. (**A**) The result achieved on the *all-patients* dataset (i.e., maximum accuracy of 72.53% combining the first 6 ranked features). (**B**) The result achieved on the *hSc* dataset (i.e., maximum accuracy of 81.25% combining the first 11 ranked features). (**C**) The result achieved on the *1-y* dataset (i.e., maximum accuracy of 93.94% combining the first 10 ranked features).

The results of the SVM-RFE automatic classification between ALS and LMND patients using the different datasets described in “Data collection” section are shown in Fig. 2. Considering the whole group of patients (*all-patients* dataset), we achieved maximum recognition accuracy of 72.53 %. This accuracy was obtained by selecting only the first 6 most informative analytes according to the RFE criterion (Fig. 2A). Taking into account only those patients at an early stage of the disease (i.e., *hSc* and *1-y* datasets), the maximum accuracy increases to 81.25 % for the *hSc* dataset using 11 analytes (Fig. 2B), and to 93.94% for the *1-y* dataset combining the first 10 ranked features (Fig. 2C).

*Most informative selected analytes* Exploring the analytes selected by the RFE algorithm (see Table 2), it is worthwhile noting that Cre, Tran, P, Ca are in the first positions among the selected analytes in the three classifications. However, the most informative ranked analytes are the immunological ones: 3 out of 6 considering the *all-patients* dataset, i.e., monocytes%, IgM, and CD3 lymphocyte counts; 5 out of 11 considering the *hSc* group, i.e. IgE, IgM, absolute leucocyte counts, CD4 and CD8 lymphocyte; 5 out of 10 considering the *1-y* group, i.e., IgE, IgG, $\gamma$Gl, CD4 and CD8 lymphocyte counts.

*Test set evaluation* The generalization performance of the classifier fitted on the *all-patients* dataset was assessed also on the test set of 11 patients described in “Generalization performance evaluation”. The result revealed an accuracy of 70.59%, i.e., consistent with the performance estimated by the LOSO procedure.

**Table 2:** List of significantly different blood analytes between ALS and LMND, and the related descriptive statistics (median and median absolute deviation, MAD).

Analyte	Median ± MAD (ALS)	Median ± MAD (LMND)	*p* values
Dataset: all-patients			
IgG	1190 ± 206.5	1000 ± 161.5	7.92E−18
MCH	30.2 ± 1.2	28.9 ± 1	6.96E−17
$\gamma$Gl	16.25 ± 2.65	13.8 ± 2.2	7.39E−17
$\alpha$2Gl	9.5 ± 0.9	11 ± 1.5	8.58E−17
CD19	9.7 ± 2.6	12.2 ± 2.5	1.69E−15
GPX	40.75 ± 10.7	30.2 ± 8.1	2.75E−15
$\beta$Gl	6.1 ± 0.6	7 ± 1	2.25E−14
P	3.1 ± 0.4	2.6 ± 0.48	1.92E−11
IgM	107 ± 36	69 ± 31	3.83E−11
MCV	89.45 ± 3.05	87.7 ± 2.6	1.76E−10
Lip	28 ± 7	35 ± 9	6.20E−10
FGF	1.6 ± 0.9	2.5 ± 1.4	1.85E−09
Chol	177.5 ± 30.5	199 ± 23	2.70E−09
Bil	0.69 ± 0.26	0.5 ± 0.11	5.10E−09
IL12	104 ± 49	158 ± 70	5.01E−08
VCAM1	856 ± 253.5	1077 ± 401	6.80E−08
RBC	4.465 ± 0.325	4.78 ± 0.26	8.45E−08
MMP9	556 ± 261.5	784.5 ± 203.5	1.43E−07
CD3	75.45 ± 4	72.3 ± 4.1	1.72E−06
MON%	6.85 ± 1.25	5.8 ± 1.2	4.62E−06
IL6R	141 ± 29	162 ± 43	9.42E−06
TNFR2	3.35 ± 1.25	4.2 ± 1.7	1.05E−05
Fer	147 ± 103	65 ± 51	1.84E−05
IL2R	1.5 ± 0.5	2.2 ± 0.9	7.08E−05
Gluc	85 ± 7	90 ± 10	7.10E−05
IL3	4.55 ± 3.05	2.6 ± 2	1.26E−04
CD4	45.9 ± 4.5	41.8 ± 3.7	2.45E−04
Fe	88 ± 22	80 ± 16	2.72E−04
IgA	196 ± 53.5	189 ± 84	2.95E−04
IGF1	133 ± 44.75	146 ± 44	8.11E−04
PLT	216 ± 59.5	245 ± 35	8.74E−04
MCHC	33.3 ± 0.7	32.8 ± 0.8	9.01E−04
NEU%	62.7 ± 4.85	65.4 ± 4.4	1.20E−03
TNFR1	1.7 ± 0.5	2.1 ± 0.6	1.27E−03
Tran	227 ± 23	241 ± 37	1.83E−03
NEU	3.99 ± 0.83	4.59 ± 0.99	2.44E−03
CK	105.5 ± 43.5	78 ± 46	2.49E−03
AST	23 ± 5	21 ± 4	2.81E−03
Trig	110 ± 34	139 ± 63	4.24E−03
IgE	36.5 ± 12.5	26 ± 11	8.47E−03
GGT	19 ± 6	23 ± 11	8.47E−03
PTT	29.9 ± 1.9	30.9 ± 1.9	9.57E−03
IL8	2.4 ± 1.1	1.8 ± 1	1.20E−02
ICAM1	295 ± 80	338 ± 79	1.41E−02
K	3.89 ± 0.18	3.96 ± 0.21	1.74E−02
LA	11.95 ± 3.35	12.8 ± 3.4	2.91E−02
TNF	5.6 ± 3.8	9.1 ± 6.5	4.06E−02
Dataset:hSc			
Tran	221 ± 19.5	270 ± 40	1.89E−05
CD8	25.9 ± 5.5	33.4 ± 5	4.03E−04
P	3.1 ± 0.4	2.4 ± 0.53	8.51E−04
IL3	5.1 ± 3.95	1.3 ± 1.1	0.0013
$\beta$1Gl	5.85 ± 0.55	7.3 ± 2	0.0028
Fer	209 ± 133	61 ± 53	0.0035
Ca	9.4 ± 0.5	8.9 ± 1.1	0.0035
Fe	86 ± 16.5	73 ± 18	0.0196
CD25	2.1 ± 0.6	3.1 ± 1.2	0.0197
CD4	47.75 ± 4.45	42.5 ± 5.7	0.0239
GPX	38.55 ± 9.55	28.9 ± 7.6	0.0239
MCH	29.85 ± 1.05	29 ± 1.1	0.0441
Dataset: 1-y			
IL2R	1.5 ± 0.45	2.3 ± 0.9	0.0235

**Table 3:** List of the most informative features selected by the SVM-RFE for each ALS/LMND classification.

Dataset	All-patients	hSc	1-y
Accuracy	72.53%	81.25%	93.94%
Selected feature ranking	Cre	P	Chol
	Tran	Ca	CD8
	MON%	WBC	IgG
	$\alpha$2Gl	CD8	$\gamma$Gl
	IgM	MCHC	HDL
	CD3	PT	PT
		IgM	Trig
		MCH	CD4
		IgE	IgE
		CD4	PTT
		SelE	

### Low versus high progression rate statistical comparison

Table 3 shows the results of the statistical comparison between patients with high and low PR, for the ALS and LMND datasets, respectively.

#### ALS dataset

In the fast progressive ALS group, we found higher levels of Ca, K, Mg, P, Vit B12, Fol, Chol HDL, ESR, and Amy than in the LMND group. On the other hand, in slowly progressing ALS, we found more basophils (BAS), a higher percentage of CD3 and CD8 cells, and higher levels of LDH, albumin, IgG, IL4, IL10, IL2R, IL6R, EPO, ICAM1, ERS, associated with lower percentages of CD16+56 and CD45 cells.

#### LMND dataset

Considering the LMND group, we observed higher levels of VitB12, Fer (associated with lower values of Tran), ALT, GGT, and LDH in the fast progressive LMND group than in the slow progressive one. Moreover, the fast progressive LMND showed also a reduced quantity of MON, EOS, TPAO, ESR, K, and Na; whereas the soluble IL2R and IGF1 resulted higher than in the slow progressive one.

### Low versus high progression rate classification results

**Figure 3:**
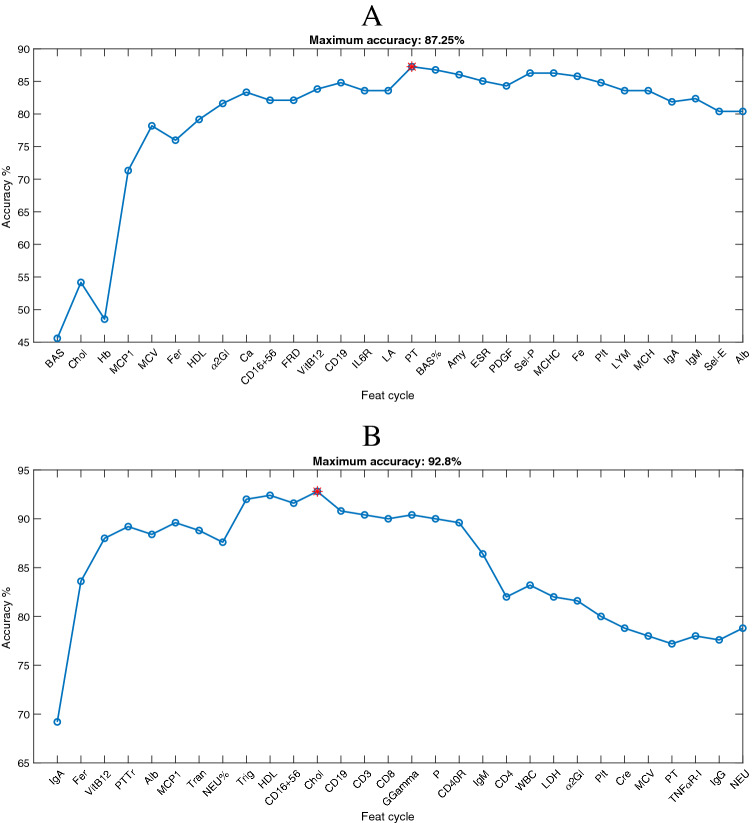
Classification accuracy trend of the low versus high progression rate recognition model as a function of the first 30 selected blood analytes. The red marker indicates the maximum accuracy. (**A**) The result achieved on the ALS dataset (i.e., maximum accuracy of 87.25% combining the first 16 ranked features). (**B**) The result achieved on the LMND dataset (i.e., maximum accuracy of 92.80% combining the first 12 ranked features).

Concerning the automatic recognition of fast and slow progressive ALS and LMND patients, high accuracy was achieved for each of the two groups. Particularly, considering the ALS patients, we obtained 87.25% of accuracy by using the first 16 ranked analytes (Fig. 3A). Likewise, considering the LMND patients, we achieved an accuracy of nearly 93 % by using the first 12 ranked analytes (Fig. 3B).

*Most informative selected features* The list of selected analytes is shown in Table 2. In both groups, the highest recognition accuracy was achieved by a combination of analytes of different origins. Interestingly, Chol, HDL, Fer, VitB12, CD16+56, and MCP1 are shared between the ALS and LMND datasets.

*Test set evaluation* The results of the generalization performance assessment on the test set showed an accuracy of 81.25% for the ALS group and 90.91% for the LMND one, confirming the very good performance estimated by the LOSO procedure.

**Table 4:** List of significantly different blood analytes between high and low PR patients within the ALS and LMND groups.

ANALYTE	Median ± MAD (Low PR)	Median ± MAD (high PR)	*p* values
*ALS patients*			
Ca	9.3 ± 0.3	9.7 ± 0.3	1.74E−19
Chol	161 ± 37	202 ± 25	4.72E−18
PT	94 ± 7	104 ± 7	1.01E−14
INR	1.04 ± 0.06	0.99 ± 0.04	1.59E−13
$\alpha$2Gl	9.1 ± 0.8	10.2 ± 1	1.49E−12
Mg	1.93 ± 0.17	2.11 ± 0.14	2.14E−10
P	2.9 ± 0.5	3.3 ± 0.3	1.21E−09
VitB12	422 ± 115	530 ± 135	1.65E−09
HDL	48 ± 11	56 ± 10	2.28E−09
VES	10 ± 8	24 ± 13	1.21E−06
PLT	195 ± 68	244 ± 44	2.92E−06
$\alpha$1Gl	3.9 ± 0.6	4.4 ± 0.5	1.05E−05
CD45	98.8 ± 0.3	99.1 ± 0.3	1.32E−05
BAS%	0.5 ± 0.2	0.4 ± 0.1	1.83E−05
CD8	29.5 ± 4.7	23.9 ± 5.5	3.16E−05
K	3.82 ± 0.19	3.96 ± 0.15	4.10E−05
MCH	30.6 ± 1.1	29.6 ± 1.1	0.00044
IL6R	149 ± 27	122 ± 28	0.0005
BAS	0.03 ± 0.01	0.02 ± 0.01	0.00078
ERS	0.31 ± 0.233	0.179 ± 0.105	0.00121
IL10	3.3 ± 2.1	2.2 ± 0.9	0.00194
Fol	6 ± 2.4	7.5 ± 2.9	0.00196
FRD	333 ± 64	295 ± 53	0.00215
IL4	0.5 ± 0.3	0.4 ± 0.1	0.00517
Alb	60.2 ± 2.9	57.6 ± 3.6	0.00525
IGG	1220 ± 200	1067 ± 208	0.00534
CD16 +56	11.8 ± 2.6	15.4 ± 5.7	0.00629
PTT	30.2 ± 2.2	29.5 ± 1.5	0.01372
BA	6 ± 1.9	7 ± 2.1	0.01568
MCV	90.3 ± 3.3	89.1 ± 2.8	0.02522
Cl	103 ± 2	102 ± 2	0.02843
ICAM1	318 ± 80.5	270 ± 60	0.03073
LA	11 ± 3.5	12.4 ± 2.1	0.03414
CD3	76.5 ± 3	72.9 ± 5.9	0.03414
EPO	12.6 ± 5.3	9.7 ± 2.3	0.03554
LDH	213 ± 57	185 ± 26	0.03556
IL2R	1.6 ± 0.6	1.4 ± 0.4	0.03556
Amy	50 ± 16	59 ± 16	0.04246
PTTr	1.01 ± 0.06	1 ± 0.05	0.04774
*LMND patients*			
Fer	49.5 ± 31.5	264.5 ± 100	1.41E−20
VitB12	432 ± 100	750 ± 235	6.04E−12
Tran	269 ± 46	213 ± 17.5	7.02E−12
MCHC	32.6 ± 0.7	34.1 ± 0.9	9.35E−11
IL2R	1.9 ± 0.7	3.8 ± 1.55	4.64E−08
PTTr	1.05 ± 0.05	1 ± 0.07	2.63E−07
MON%	6.2 ± 1	4.5 ± 1	1.24E−06
K	4.03 ± 0.22	3.79 ± 0.16	4.12E−06
ALT	19 ± 6	28 ± 8	3.63E−05
EOS%	2.5 ± 1.1	1.45 ± 0.45	1.15E−04
IGF1	132 ± 34	183 ± 51	1.13E−04
PTT	31.2 ± 1.6	29 ± 2.15	1.19E−04
PLT	250 ± 30	219 ± 38.5	5.67E−04
EOS	0.16 ± 0.05	0.11 ± 0.04	0.00157
MON	0.4 ± 0.08	0.325 ± 0.075	0.00159
NEU%	64.55 ± 4.35	68.4 ± 4.95	0.00314
HCT	41.9 ± 2.2	38.8 ± 2.2	0.00444
GGT	19.5 ± 7.5	59 ± 44	0.01028
TPAO	1.085 ± 0.2	0.96 ± 0.205	0.01072
Na	140 ± 1	139 ± 2	0.03027
ERS	22 ± 7.5	14.5 ± 11.5	0.04039
Bil	0.5 ± 0.1	0.605 ± 0.17	0.04039
LDH	173 ± 43.5	213 ± 37.5	0.049

**Table 5:** List of the most informative features selected by the SVM-RFE for each High PR/Low PR classification problem.

Patient group	ALS	LMND
Accuracy	87.25%	92.8
Selected feature ranking	BAS	IgA
	Chol	Fer
	Hb	VitB12
	MCP1	PTTr
	MCV	Alb
	Fer	MCP1
	HDL	Tran
	a2Gl	NEU%
	Ca	Trig
	CD16+56	HDL
	FRD	CD16+56
	VitB12	Chol
	CD19	
	IL6R	
	LA	
	PT	

## Discussion

In our study, we introduce a novel dataset of blood data and present an ML approach aiming at supporting clinicians in making a differential diagnosis of MNDs. Specifically, the applied learning algorithm is able to discriminate ALS from LMND patients, using blood data information exclusively. Moreover, our approach is able to predict the prognosis of MND patients with remarkable accuracy, recognizing whether the patients have high or low disease progression. Our results are obtained performing an automatic selection of the best combination of blood analytes ensuring the maximum classification accuracy.

Over the last 10 years, we have enrolled 55 ALS and LMND patients and collected 692 blood samples from which 108 blood parameters have been extracted. This outstanding collection of blood analytes together with a large number of blood samples is unique in the scientific literature and grants an important value to our results. Moreover, most of the studies focus on more invasive and expensive methods, such as CSF analysis or neuroimaging, not suitable for repeated sampling over time, rather than routine investigations. Indeed, plasma, easily available, represents an attractive biological fluid for the detection of biomarkers, and extensible CSF-based biomarkers^34^. ML models and large datasets offer unprecedented opportunities to appraise candidate diagnostic, monitoring, and prognostic biomarkers^17^. Our database has been used as input of an SVM-RFE algorithm. This method together with the LOSO cross-validation strategy allows mitigating the risk of confounding classification results (i.e., overfitting), which cannot be underestimated with such a number of features (i.e., 108). Indeed, on the one hand, the LOSO strategy reduces the risk of a biased optimistic estimation of the classifier accuracy avoiding the presence of observations of the same subject in both the training- and test-set. On the other hand, the RFE algorithm reduces the dimension of the dataset and, at the same time, selects the combination of analytes that maximizes the accuracy using the SVM classifier. To our knowledge, this is the first study, which investigates and develops characterization and classification of different MNDs (ALS and LMND) at single-subject level, based on blood data alone. In addition, a better understanding as well as an early recognition and prognosis of ALS and LMND may have a significant impact on research activities concerning not only the differential diagnosis but also the development of specific differentiated treatment of ALS and other MNDs. To this end, given the little progress that has been made in these last years, a novel system able to support the clinical practice is highly desirable. Our results showed a good prediction accuracy (72.53%) in recognizing the disease form of the patient under examination (ALS vs. LMND) that even strongly increased when the early stage of the disease was considered (81.25% based on the ALSFRSR, and 93.94% considering the first year of the disease). The three patients’ subgroups are associated with different combinations of blood parameters (Table 2), which allow discriminating between ALS and LMND with the highest accuracy. On the one hand, our selected analytes confirmed the importance of blood immunological properties in the discrimination of MNDs, as already reported by Lu et al^23^. In fact, we found that, among the selected analytes, most were inflammatory and immunological. Accordingly, some of the most relevant information was found in the leukocytes and their related analytes (i.e., lymphocyte subsets and immunoglobulins). Some of these analytes were highlighted also in the univariate statistical analysis. Particularly, ALS as compared with LMND patients were characterized by increased percentages of CD3, CD4, and CD8, as already observed in ALS patients compared to healthy controls^35^, as well as higher levels of IgM. On the other hand, our classifier revealed the importance of some non-standard predictive features such as P, Cre^36^, and Tran^37^, which have been only recently indicated as factors related to the disease and potential markers and are still under study. Such data could highlight important new mechanisms related to the disease. Moreover, our statistical results have indicated significant differences in other recently proposed non-standard potential markers such as RBC, MCH, and MCV^38^, ICAM1 and VCAM1^39^, FGF and IGF1^40^, and MMP9^41^. Future investigations on them might have a strong impact on translational medicine, helping to provide early diagnosis of MNDs. Concerning the disease progression, by means of an ML approach, we succeeded in classifying slowly versus fast progressive ALS and LMND patients with very good prediction accuracy (87.25% and 92.8%, respectively), indicating the potential of blood analyte measurements for prognostic purposes. Exploring the selected blood analytes for the evaluation of prognosis, we found that the RFE algorithms were able to select a common group of markers for both diseases: VitB12, CD16+56, Chol, HDL, and Fer. As far as VitB12 is concerned, no correlation data of its endogenous levels to disease severity have been reported. CD16+56 has been found higher in ALS patients compared to healthy controls^35^. Contrasting results are still reported for Fer^42^ or Chol and HDL^43^ as biomarkers, nevertheless, some studies suggest that hyperlipidemia is a protective factor in ALS^13^. This could suggest that the aforementioned analytes play a crucial role in differentiating the disease progression regardless of the type of MNDs. More in detail, from the statistical comparison we observed that VitB12 was significantly higher in fast versus slow progressive patients for both ALS and LMND groups. Whereas, on the one hand, significantly higher amounts of Chol and HDL characterized fast PR in ALS patients exclusively, on the other hand, higher Fer levels were related to fast PR in the LMND group only. Of note, since the resulted optimal learning model only requires the acquisition of few blood analytes, some of them typical of routine clinical analysis, not only the risk of overfitting is strongly mitigated, but this leads to a diagnosis and prognosis support tool with reasonably low costs.

Due to the difficult and the slow process of recruiting such kinds of patients, the high economic cost for the biochemical analyses, and the strict inclusion criteria, the patient sample size is limited, even if the number of blood samples is large. Moreover, when the *hSc* and *1-y*, as well as the PR classification problems are considered, the datasets are subjected to a decrease in the number of observations. This might induce a higher risk of overfitting. For this reason, the applied methodological strategies were specifically conceived to mitigate the risks due to a non-large number of recruited patients and to make our 692 observations enough to achieve positive, robust, and replicable results. It is worthwhile noting that even considering the prediction accuracy achieved after selecting only the first five most informative features, and consequently reducing the complexity of the model and the overfitting risk, recognition accuracy of over 75% was always reached in all classification tasks, except for the *hSc* problem where 6 features were necessary. Moreover, to test the generalization performance of the proposed recognition systems, and, therefore, the possibility to export our results in a real clinical scenario, we tested the fitted model on a test set including 11 new patients. The results confirm even in this case very high accuracy consistent with that estimated during the LOSO procedure. This is a further confirmation of the robustness of our recognition system suggesting good replicability of our results, and the fact that the relatively low amount of data did not strongly affect the reliability of the results.

In conclusion, this study, besides strengthening the importance of the immunological components in the MNDs diseases, raises many questions about those analytes (widely used but trivial) that have shown to be important in the discrimination of ALS and LMND but not yet specifically related to the different types of MNDs. On the other hand, the immunological information is not sufficient if it is not supported by other blood analytes that so far have been considered non-standard markers for neurodegenerative diseases. Moreover, our data and results strongly support the hypothesis that ALS and LMND represent two different diseases, whereas in many cases they are considered and treated as a single one.

Although significant p-values were reported for several analytes, the confidence intervals (Median ± MAD) should not be translated into a list of cut-offs levels to be used in the clinical practice. Indeed, despite the statistical significance, such intervals are often strongly overlapped between the two groups under comparison as well as fall within the ranges of healthy controls. On the other hand, our classification system might provide the clinicians with an automatic tool that can easily support the differential diagnosis of the LMND and ALS patients, showing the resulted class with the related accuracy level, in an easier and more interpretable way compared to the statistical cut-offs. It is also surprising to note that the accuracy increased when data related to the first year from the onset of the symptoms are considered. Consequently, our results can support the clinician in differentiating between the two diseases at the very early stage of the disease, whereas, with the normal clinical practice, it is often difficult to understand the actual involvement of the upper motor neuron.

From the methodological point of view, this study does not add a significant innovation in the machine learning field, although the selected method perfectly fits the aims of our study and the specifications of our type of data. However, this study can be considered as an onset for future innovative methodological applications. Indeed, data collection will go on to increase the number of patients and blood samples. This will give the possibility to apply deep learning-based classification methods, which might lead to further improvement of the classification performance.

To sum up, this work introduces a new tool to apply automatic techniques for the diagnosis and prognosis of different MNDs and paves the way for future research in which clinicians and scientists will search for an effective treatment for MNDs following a differential and selective approach. Our next study will deeply investigate these analytes that have been automatically selected using a data-driven approach and will compare these results with those achieved including some a priori clinical knowledge in the learning models. Moreover, hierarchical regression models will be employed to predict the disease progression at a single-subject level.
